# Nitric Oxide Increases Arterial Endotheial Permeability through Mediating VE-Cadherin Expression during Arteriogenesis

**DOI:** 10.1371/journal.pone.0127931

**Published:** 2015-07-02

**Authors:** Baolin Yang, Baizhen Cai, Panyue Deng, Xiaoqiong Wu, Yinglu Guan, Bin Zhang, Weijun Cai, Jutta Schaper, Wolfgang Schaper

**Affiliations:** 1 Department of Histology & Embryology, School of Basic Medicine, Central South Univ., Changsha, 410078, Hunan, P.R. China; 2 Dept. of Intensive Care Unit, the 3rd Xiangya Hospital, Central South Univ., Changsha, 410013, Hunan, P.R. China; 3 Department of Anatomy & Neurobiology, School of Basic Medicine, Central South Univ., Changsha, 410013, Hunan, P.R. China; 4 Department of Anatomy, School of Basic Medicine, Nanchang Univ., Nanchang, 330006, Jiangxi, P.R. China; 5 Max-Planck-Institute for Heart and Lung Research, Arteriogenesis Research Group, Bad Nauheim, D-61231, Germany; Universitätsklinikum des Saarlandes, GERMANY

## Abstract

Macrophage invasion is an important event during arteriogenesis, but the underlying mechanism is still only partially understood. The present study tested the hypothesis that nitric oxide (NO) and VE-cadherin, two key mediators for vascular permeability, contribute to this event in a rat ischemic hindlimb model. In addition, the effect of NO on expression of VE-caherin and endothelial permeability was also studied in cultured HUVECs. We found that: 1) in normal arteriolar vessels (NAV), eNOS was moderately expressed in endothelial cells (EC) and iNOS was rarely detected. In contrast, in collateral vessels (CVs) induced by simple femoral artery ligation, both eNOS and iNOS were significantly upregulated (P<0.05). Induced iNOS was found mainly in smooth muscle cells, but also in other vascular cells and macrophages; 2) in NAV VE-cadherin was strongly expressed in EC. In CVs, VE-cadherin was significantly downregulated, with a discontinuous and punctate pattern. Administration of nitric oxide donor DETA NONOate (NONOate) further reduced the amounts of Ve-cadherin in CVs, whereas NO synthase inhibitor L-NAME inhibited downregulation of VE-cadherin in CVs; 3) in normal rats Evans blue extravasation (EBE) was low in the musculus gracilis, FITC-dextron leakage was not detected in the vascular wall and few macrophages were observed in perivascular space. In contrast, EBE was significantly increased in femoral artery ligation rats, FITC-dextron leakage and increased amounts of macrophages were detected in CVs, which were further enhanced by administration of NONOate, but inhibited by L-NAME supplement; 4) *in vitro* experiments confirmed that an increase in NO production reduced VE-cadherin expression, correlated with increases in the permeability of HUVECs. In conclusion, our data for the first time reveal the expression profile of VE-cadherin and alterations of vascular permeability in CVs, suggesting that NO-mediated VE-cadherin pathway may be one important mechanism responsible, at least in part, for macrophage invasion during arteriogenesis.

## Introduction

Monocyte/macrophage plays an important part during arteriogenesis. It is because monocytes/macrophages are the main source of growth factors and cytokines such as basic fibroblast growth factor (bFGF) or TNFα, which contribute to collateral growth and remodelling[1]. Furthermore monocyte-deficient op/op mice showed only a stunted collateral response to femoral artery occlusion, whereas intravenous injections of blood-isolated monocytes could rescue the flow recovery in 5-fluorouracil induced monocyte-deficient rabbits [2, 3]. As early as 1976 Schaper and Schaper showed that monocytes adhere, migrate into deeper parts of the vessel wall, and/or populate the adventitial space [4]. Later, numerous immunohistochemical experiments confirmed this finding [1, 5]. After many years’ study, it is well-known that the mechanism for the adhesion of monocytes to the endothelial lining involves a panel of different adhesion and chemokine molecule groups produced by activated endothelial cells under increased shear stress, such as ICAM and MCP-1, but the question how monocytes transmigrate through the endothelial barrier into underlying tissue remains open.

The barrier of the endothelial lining consists of cell-to-cell contacts including tight junction and adherens junction, that control critical endothelial functions both in quiescent conditions and in activated situations such as inflammation and angiogenesis [6,7]. Vascular endothelial-cadherin (VE-cadherin), the transmembrane component of the endothelial adherens junction in all types of vascular endothelial cells, is involved in the maintenance of cell-to-cell contacts [8]. In vitro experiments showed the permeability is decreased in VE-cadherin overexpressing cells[9], whereas endothelial monolayers treated with antibodies against VE-cadherin display an increases in permeabitity[10]. Furthermore, Corada et al showed in vivo administration of a monoclonal antibody against VE-cadherin induces an increase in vascular permeability in heart and lungs [11]. In addition, Taddei et al reported that endothelial adherens junctions control tight junctions by VE-cadherin-mediated upregulation of claudin-5[12]. These experiments indicate VE-cadherin plays a pivotal role in endothelium integrity and in the control of vascular permeability. However no information is available till now about whether VE-cadherin is involved in the invasion of monocytes/macrophages and collateral vessel growth during arteriogenesis.

The function and organization of VE-cadherin can be regulated by numerous vasoactive agents such as histamine, prostaglandins, thrombin and nitric oxide[13–15]. Of these, nitric oxide may be particularly important, because on one hand NO donors (SIN-1 and SNAP) markedly reduced the amount of VE-cadherin in murine microvascular endothelial cells, accompanying an increase in vascular permeability[15]; On the other hand, the number of monocytes/macrophages in iNOS-/- and eNOS-/- mice was reduced during arteriogenesis, but recovered after the supplement of NO donor, diethylenetriamine NONOate (DETA NONOate) [16]. Therefore, we hypothesized that NO-mediated VE-cadherin pathway may be an important mechanism for the invasion of monocytes/macrophages during arteriogenesis. To test this hypothesis, we first used immunoconfocal microscopy with specific antibodies to determine the relationship between the expression of VE-cadherin and macrophage invasion in collateral vessels (CVs) in rat hind limb induced by femoral ligation with or without the treatment of either DETA NONOate or the NO synthase inhibitor L-NAME. Next, we evaluated the endothelial permeability by checking Evans Blue extravasation (EBE) in the musculus gracilis and fluorescein isothiocyanate-dextran (FITC- dextran) leakage in CVs with or without the treatment of either DETA NONOate or L-NAME. Finally the effect of NO on expression of VE-cadherin and endothelial permeability in cultured human umbilical vein endothelial cells (HUVECs) was investigated.

## Methods

### Animal model

The experimental protocol was approved by the Animal Care and Use Committee of Central South University and conformed to the National Institutes of Health Guide for the Care and Use of Laboratory Animals. Seventy two adult Sprague-Dawley (SD) rats were used in this study. They were subdivided into 3 groups: simple femoral artery ligature (FAL, n = 24), femoral artery ligature plus DETA NONOate (FAL+ NONOate, n = 24) and femoral artery ligature plus L-NAME (FAL+ L-NAME, n = 24). In each group, 8 rats were used for immunostaining study, 8 rats for modified miles vascular permeability assay, 8 rats for fluorescent dextran injection. For sham control, sham and FAL operation were performed in the same rat, the left side for sham, the right side for FAL. All experimental animals were provided by the Animal Center, Xiangya School of Medicine, Central South University. The animals were anesthetized with sodium pentobarbital (50 mg/kg, intraperitoneally). The right femoral artery ligature was performed with two knots. Then the skin was closed with sterile surgical clips. The animals were allowed to recover completely, and housed with free access to water and food. DETA NONOate and L-NAME (Sigma-Aldrich) were injected to the rats intraperitoneally at a dose of 2.5 mg/kg and 40 mg/kg body weight/day, respectively for seven consecutive days. For simple femoral artery ligature rats, only normal saline were injected. Gangrene and gross impairment of hind limb function were not observed in all the rats.

### Tissue sampling and immunohistochemistry

At day 7 post-surgery, The animals were sacrificed under overdose of anesthesia with sodium pentobarbital (80 mg/kg, intraperitoneally). Gracilis muscles were removed for exepiments. By our previous experience, this muscle contains 1–2 collateral vessels constantly during arteriogenesis. A total of 48 vessels were investigated. All samples were immediately frozen in liquid nitrogen, embedded in tissue processing medium (O.C.T) and stored at -80°C till further use. Cryosections were cut 5-μm thick, fixed in 4% paraformaldehyde, then pre-incubated in 0.2% BSA-C (Aurion Co.) and thereafter incubated with the primary antibodies (Table 1). Incubation of second antibodies (Table 1) at a concentration of 1:200 was followed by Cy2 conjugated Streptavidin (Biotrend). The nuclei were stained with TOTO3 (Molecular Probes). The sections were coverslipped and viewed with a Leica confocal microscope (Leica TCS SP). Further documentation and image analysis were carried out using a Silicon Graphics Octane workstation (Silicon Graphics) and three-dimensional multichannel image processing software (Bitplane).

**Table 1:** Primary and secondary antibodies used in this study.

Antigen	Clone	Host	Dilution	Company
Ve-cadherin		Rabbit	1:100	US biological Co
eNOS	Clone3	Mouse	1:100	BD Transduction
iNOS		Mouse	1:100	BD Transduction
CD11b		Mouse	1:100	Chemicon USA
Actin-Phallodin	TRITC labeled			Sigma
Anti-rabbit-IgG		Donkey	1:100	Dianova
Anti-mouse-IgG		Donkey	1:100	Dianova

Immunostaining for cultured cells was performed following a similar protocol as described above, except for primary antibody incubation that was conducted at 4°C overnight. The nuclei were stained with 7-aminoactinomycin D.

### Fluorescent dextran injection

Dextran coupled with FITC (FITC-dextran, Sigma-Aldrich) was chosen to assess the alterations of arterial endothelial permeability of collateral vessels. Rats were injected via the tail artery with 0.25 ml of 5% FITC-dextran per 100 g body weight 2 hours before they were killed. The musculus gracilis was removed and cryosections were cut 5-μm. Then immunofluorescent staining for CD31 (endothelial marker, detected by cy3 fluorescein) was performed. The sections were viewed with a Leica confocal microscope (Leica TCS SP). The amount of FITC-dextran in the musculus gracilis was determined with confocal microscopy.

### Modified miles vascular permeability assay

The modified miles assay was performed as previously described [17]. Briefly, under anesthesia, the rats were injected with Evans Blue at a concentration of 2% (40 mg/kg; Sigma-Aldrich) through great saphenous vein. After 60 minutes, rats were euthanized and the musculus gracilis was removed, oven-dried at 55°C, and weighed. Evans blue was then extracted from the muscle using 1000 μl of formamide for 24 hours at 55°C. Evans blue extravasation into the muscle was measured spectrophotometrically at 630 nm using a standard curve of Evans blue in formamide.

### Measurement of nitric oxide production after supplement of DETA NONOate or L-NAME or normal saline to the rats or HUVECs

Serum was collected at day 7 from DETA NONOate or L-NAME or NaCl-treated rats for determination of NO metabolites using the Nitrate/Nitrite Colorimetric Assay Kit (Cayman Chemical Company, Cat. No. 780001). Briefly, serum was filtered through Ultra-filter devices (Amicon, Millipore, Schwalbach, Germany) at 14,000 rpm for 15 minutes to obtain protein free pure plasma. Then 40 μL of the serum or culture media from DETA NONOate or L-NAME or NaCl-treated HUVECs was diluted with 40 μL of assay buffer and mixed with 10 μL of the enzyme cofactor mixture and 10 μL of the nitrate reductase. After 3 hours of incubation to convert nitrate to nitrite, total nitrite was measured at 540 nm with Griess reagents (R1 and R2) using the microplate reader. Concentrations of nitric oxide in the samples were determined using nitrate standard curve.

### Cell Culture

Human umbilical vein endothelial cells (HUVECs, ScienCell USA) were seeded on Lab-Tek Chamber Slide (NUNC, USA) and cultured in endothelial cell medium (ScienCell USA) with 10% fetal calf serum (FCS), according to the supplier’s protocol.

### In vitro permeability assay

Endothelial permeability was evaluated in vitro by diffusing of fluorescein isothiocyanate (FITC)-dextran (Sigma) through the endothelial monolayer. Briefly, HUVECs were grown to confluence on transwell inserts (Costar, Cambridge, MA, USA). The cells were treated with different concentrations of DETA NONOate or L-NAME or simple culture media respectively for 24 hours. Then 30μl of medium containing FITC-dextran(10 mg/ml) was added to the upper compartment of the transwell. The amount of FITC-dextran that diffused through the endothelial monolayer into the lower compartment was quantified using a microplate reader (Molecular Devices, Sunnyvale, CA).

### Immunoblotting of VE-Cadherin

To study the effects of DETA NONOate or L-NAME on the protein level of VE-cadherin, HUVECs were lysed with RIPA buffer (Sigma) containing 1 mM phenylmethylsulfonyl fluoride, and the total protein was subjected to 8% SDS-polyacrylamide gel. Transferred membrane was probed with the rabbit anti-VE-cadherin antibodies (Santa Cruz Biotechnology).

### Quantitative Measurements

The quantification of immunofluorescence intensity was performed with a Leica TCS SP confocal microscope, using the quantitation software from Leica as described previously[18]. Brifely, one channel with format 512 and appropriate filters was used. A full range of gray values from black to peak white (0-pixel to 255-pixel intensity level) was set during the whole process of measurements. The intensity of fluorescence was expressed as arbitrary units AU/μm^2^. Four sections from each vessel were measured.

The quantitative analysis of CD11b positive cells was performed with the confocal microscope. The CD11b positive products with nuclear staining inside was counted as CD11b positive cells and the ratio of the number CD11b positive cells to the total number of nuclei of vascular wall was considered as macrophage index. Four sections from each vessel were measured.

All data are presented as means ± SEM. Statistical analysis of all data were performed using one-way ANOVA followed by a multiple comparion test. Statistical significance was accepted when p<0.05.

## Results

### Expression of eNOS and iNOS in collaterals of sham and femoral artery ligation (FAL) rats

**Fig 1:**
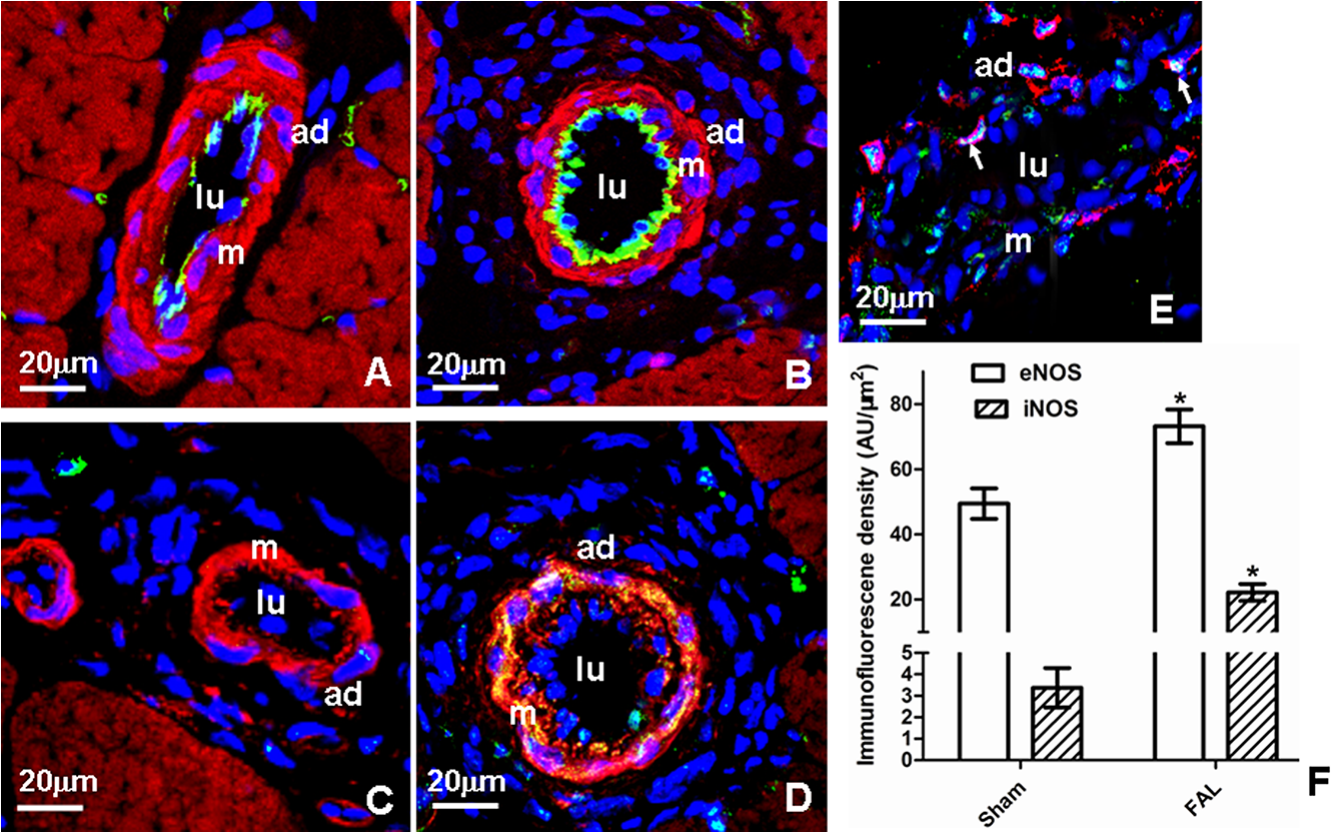
Expression of eNOS and iNOS in collaterals of sham (Sham) and femoral artery ligation (FAL) rats. A-D: eNOS and iNOS immunostaining. E: dual immunostaining of iNOS with CD11b (marker of macrophage). Specific fluorescence: green for eNOS in A and B, for iNOS in C-E; red for F-actin in A-D, for CD11b in E; blue for nuclei. A and C: Sham; C, D and E: FAL; F: quantitative analysis of immunofluorescence intensity of eNOS in SV, and FAL. Note that in FAL, both eNOS and iNOS proteins were remarkably increased, induced iNOS was observed in all the layers of the vascular wall and macrophages (E, arrowheads). Lu: lumen; m: tunica media; ad: adventitia; *P < 0.05 vs sham.

In sham rats, eNOS was moderately expressed in EC, iNOS was rarely detected (Fig 1A and 1C). In FAL rats, both eNOS and iNOS were significantly increased, 1.5-fold and 6.5 fold over that in sham group, respectively (Fig 1B, 1D and 1F). Increased iNOS was mainly distributed in SMCs, but also seen in other vascular cells (Fig 1D). Expression of iNOS in macrophages was revealed by dual immunostaining of iNOS with CD11b (Fig 1E).

### Expression of Ve-cadherin, vascular permeability and macrophages in collaterals of sham and femoral artery ligation (FAL) rats

**Fig 2:**
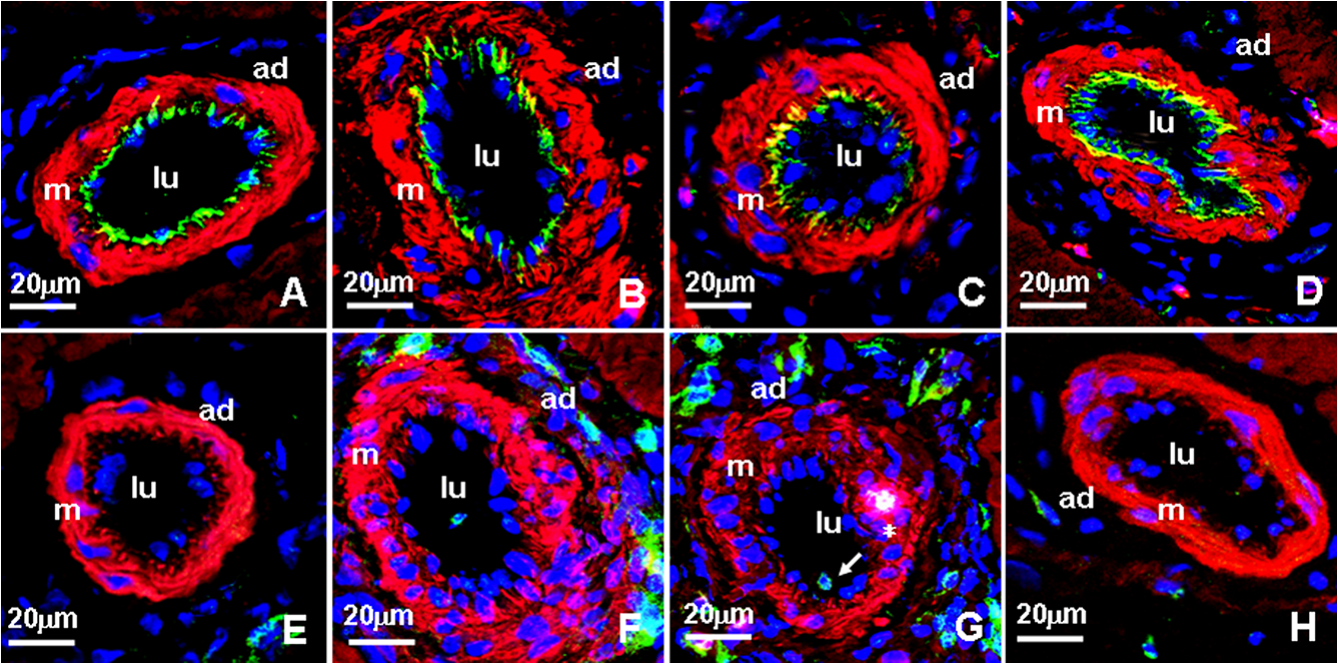
Confocal micrographs of VE-cadherin (A-D) and CD11b (E-H) immunostaining in sham (SV), femoral artery ligation (FV), NONOate treated (NNV) and L-NAME treated (LV) collateral vessels. Specific fluorescence: green, red for F-actin., blue for nuclei. A and E: SV; B and F: FV. C and G: NNVE. D and H: LV. Note that VE-cadherin was moderate in SV, decreased in FV, further reduced in NNV, strongly stained in LV; CD11 positive cells were less in SV and LV, strongly increased in NNV where they were detected in the abluminal region and the media (G, arrowheads and star).

**Fig 3:**
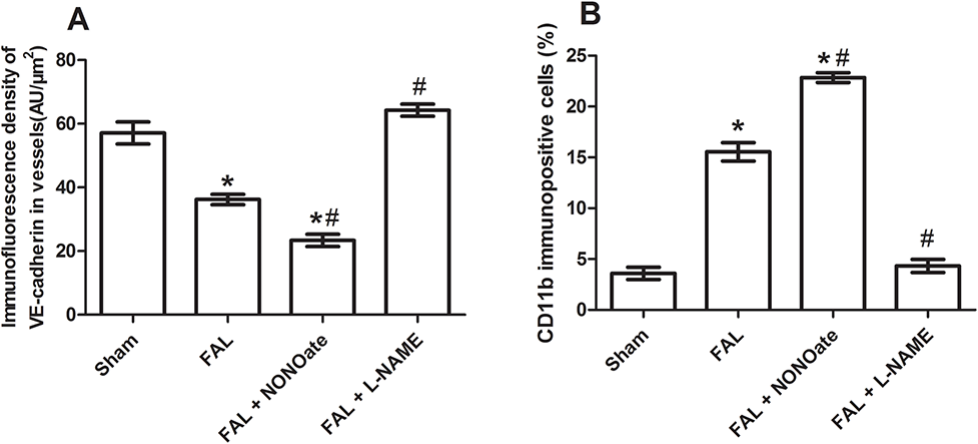
Quantitative analysis of immunofluorescence density (AU/μm2) of VE-cadherin and CD11 positive cells (%) in sham (sham), femoral artery ligation (FAL), NONOate treated (FAL + NONOate) and L-NAME treated (FAL + L-NAME) collateral vessels. *P < 0.05 vs sham., #P < 0.05 vs FLA.

**Fig 4:**
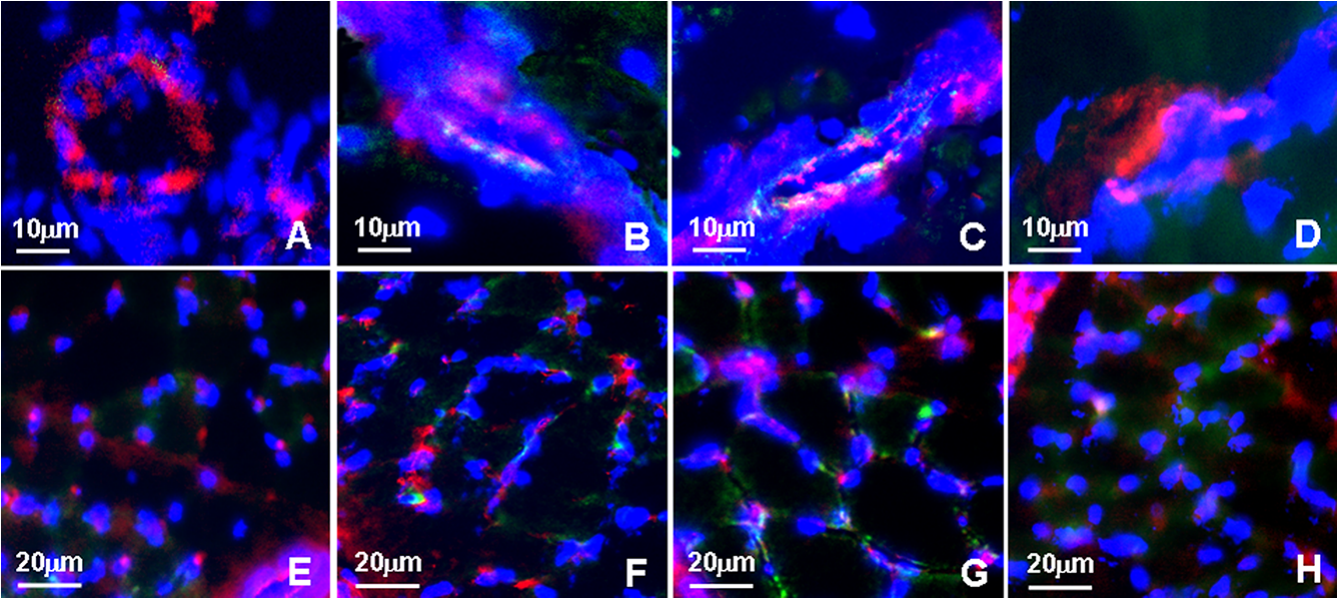
Confocal micrographs of FITC-dextran leakage in the musculus gracilisthe. Specific fluorescence: green, red for CD31(marker of endothelial cells)., blue for nuclei. A-D: collaterals, E-H: capilaries. A and E: sham, B and F: femoral artery ligation (FAL), C and G: NONOate treated, D and H: L-NAME treated. Note that FITC-dextran was not observed in sham and L-NAME treated collaterals, but detected in FAL collaterals and enhanced in NONOate treated collaterals; in capillaries level, FITC-dextran was observed in all the groups, but more in FAL and NONOate treated ones.

**Fig 5:**
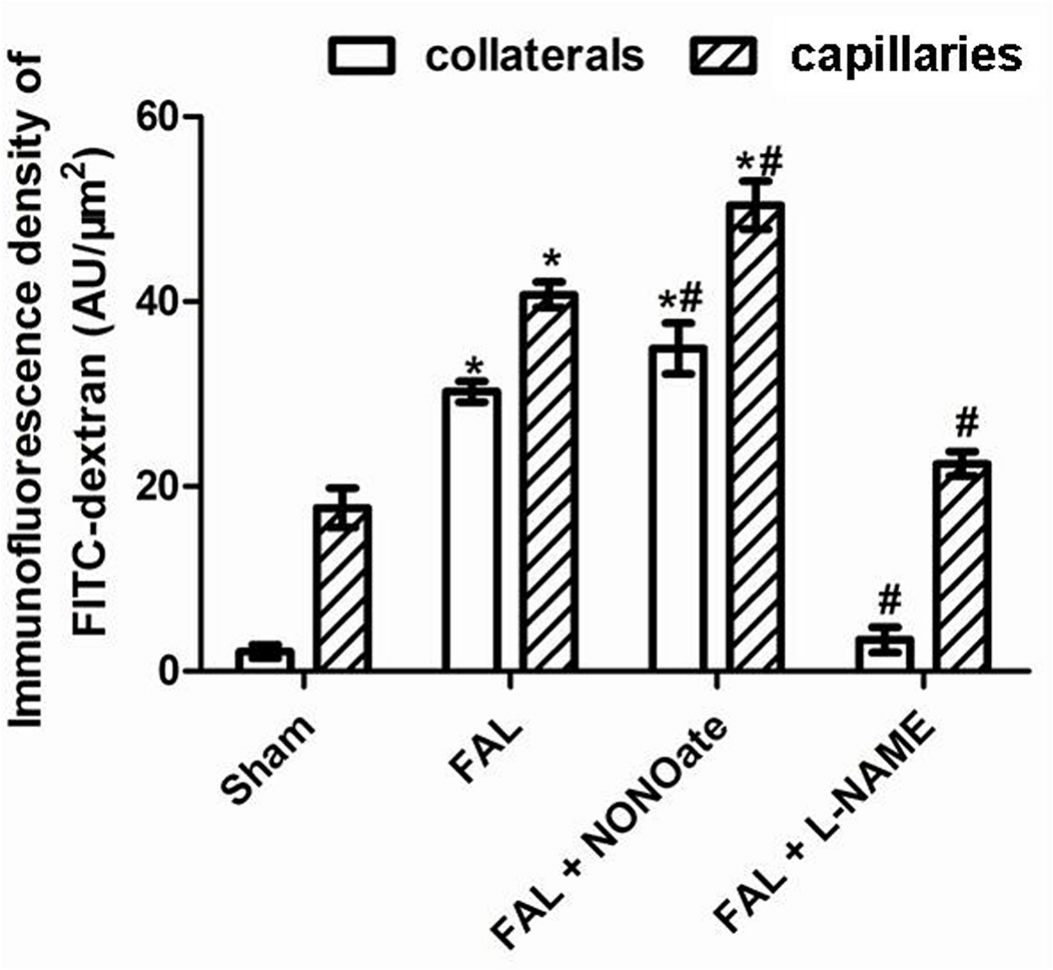
Quantitative analysis of fluorescence density (AU/μm2) of FITC-dextran in the collaterals and capilaries of the musculus gracilis from sham, femoral artery ligation (FAL), NONOate treated (FAL + NONOate) and L-NAME treated (FAL + L-NAME) groups. *P < 0.05 vs sham., #P < 0.05 vs FAL.

**Fig 6:**
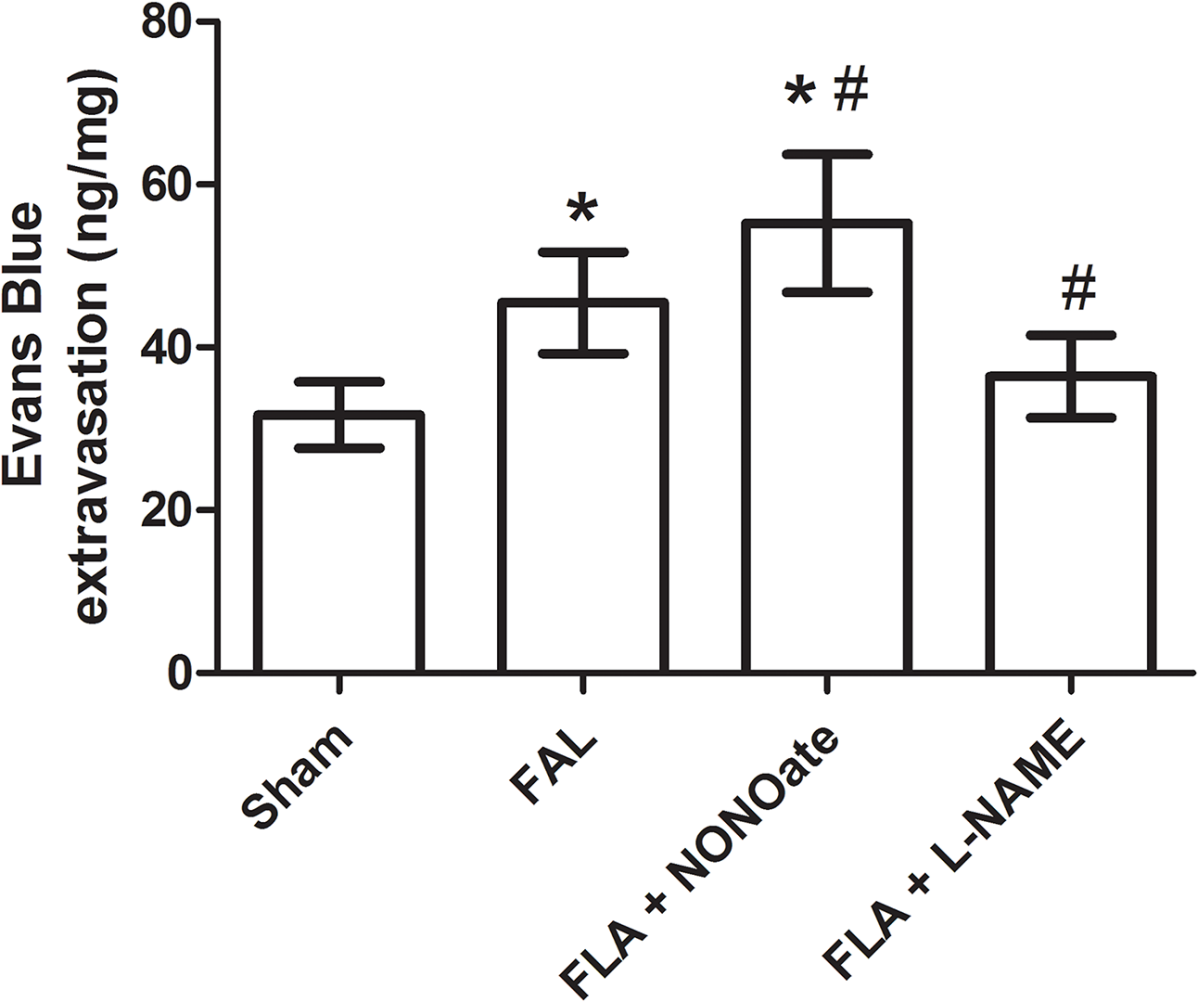
Quantitative analysis of Evans Blue extravasation (ng/mg) in the musculus gracilis from sham, femoral artery ligation (FAL), NONOate treated (FAL + NONOate) and L-NAME treated (FAL + L-NAME) groups. *P < 0.05 vs sham., #P < 0.05 vs FAL.

In sham rats, VE-cadherin was strongly expressed in EC, the staining between EC was continuous, whereas in FAL rats, VE-cadherin staining was present as discontinuous and punctate pattern (Fig 2A and 2B). Quantitative analysis of fluorescence density showed that VE-cadherin protein was significantly reduced in FAL rats (P<0.05), 1.58 fold lower than that in sham rats (Fig 3A). In the permeability test, FITC-dextran was rarely detected in the vascular wall and was very low in the musculus gracilis in sham rats (Fig 4A and 4E and Fig 5). In contrast, in FAL rats, FITC-dextran was present in the vascular wall and FITC-dextran leakage in the musculus gracilis was significantly increased (Fig 4B and 4F and Fig 5). Similar to FITC-dextran examination, Evans blue extravasation (EBE) was significantly increased in the musculus gracilis in the FAL rats than that in sham rats (Fig 6). Correlated with the changes in FITC-dextran/ EBE leakage, few macrophages were detected in sham rats, but significantly increased (P<0.05) in FAL rats (Fig 2E and 2F and Fig 3B).

### Effect of DETA NONOate or L-NAME on expression of VE-cadherin, vascular permeability and macrophage invasion during arteriogenesis

**Fig 7:**
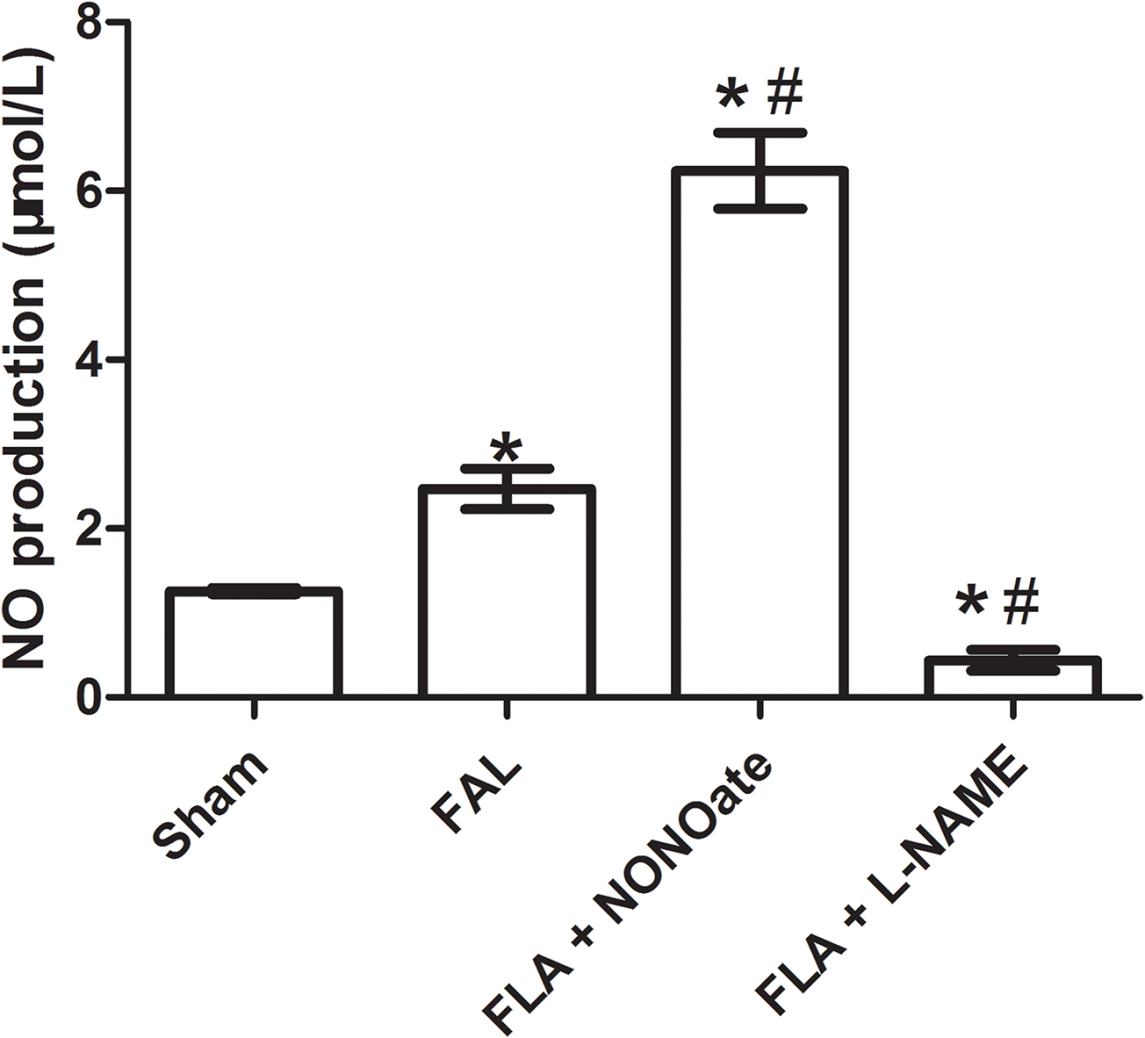
Quantitative analysis of NO production (μM/L) in the serum from sham, femoral artery ligation (FAL), NONOate treated (FAL + NONOate) and L-NAME treated (FAL + L-NAME) groups. *P < 0.05 vs sham., #P < 0.05 vs FAL.

As compared to those in the only FAL rats, VE-cadherin staining was weaker and more intermittent in the collaterals of NONOate treated FAL rats (Fig 2B and 2C), and the amount of VE-cadherin protein was further reduced, 1.5 fold lower than that in FAL rats (Fig 3A). FITC-dextran leakage and Evans blue extravasation (EBE) were significantly increased in the collaterals and capillaries of the musculus gracilis in NONOate treated rats (Fig 4C and 4G, Fig 5 and Fig 6). Coupled with the increase in vascular permeability, the number of macrophages in the collaterals in NONOate treated rats was significantly increased (Fig 2G and Fig 3B). In contrast, in collaterals of the rats treated with L-NAME, VE-cadherin staining was strong and continuous around EC (Fig 2D), VE cadherin expression was even slightly higher than that in sham rats (Fig 3A. 57.10±2.0 and 63.01±1.03 AU/μm^2^). FITC-dextran leakage was not observed in the vascular wall, FITC-dextran leakage and Evans Blue extravasation was very low, similar to that in sham rats (Fig 4D and 4H, Fig 5 and Fig 6). Correlated with these observations, few macrophages were detected in these collateral vessels (Fig 2H and Fig 3B). In addition, Colorimetric Assay confirmed that NO production was singnificantly increased in the serum from NONOate treated rats, whereas the treatment with L-NAME inhibited NO production (Fig 7).

### Effect of DETA NONOate or L-NAME on expression of VE-cadherin and EC permeability in HUVECs

**Fig 8:**
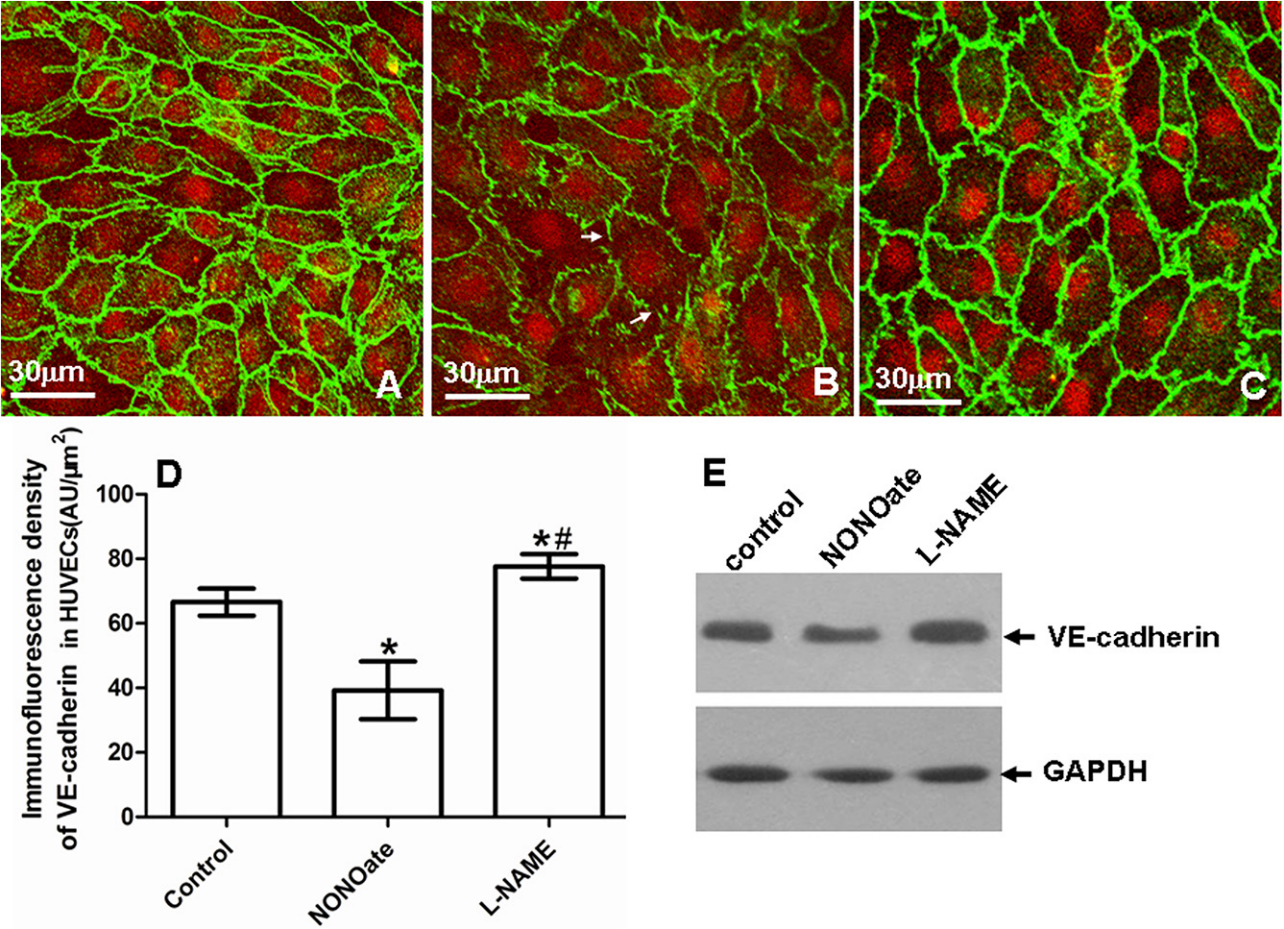
Expression of VE-cadherin in HUVECs. A-C: VE-cadherin immunostaining. A: control, B: NONOate treated, C: L-NAME treated. Specific fluorescence: green for VE-cadherin, red for nuclei. Note that in control and L-NAME treated HUVECs VE-cadherin staining was strong and continuous and distributed around the entire periphery of cells, in NONOate treated HUVECs, VE-cadherin staining was weak and intermittent (B, arrowheads). D: quantitative analysis of immunofluorescence intensity of VE-cadherin in HUVECs (*P < 0.05 vs control, #P < 0.05 vs NONOate treated or control). E: immunoblotting of VE-Cadherin.

**Fig 9:**
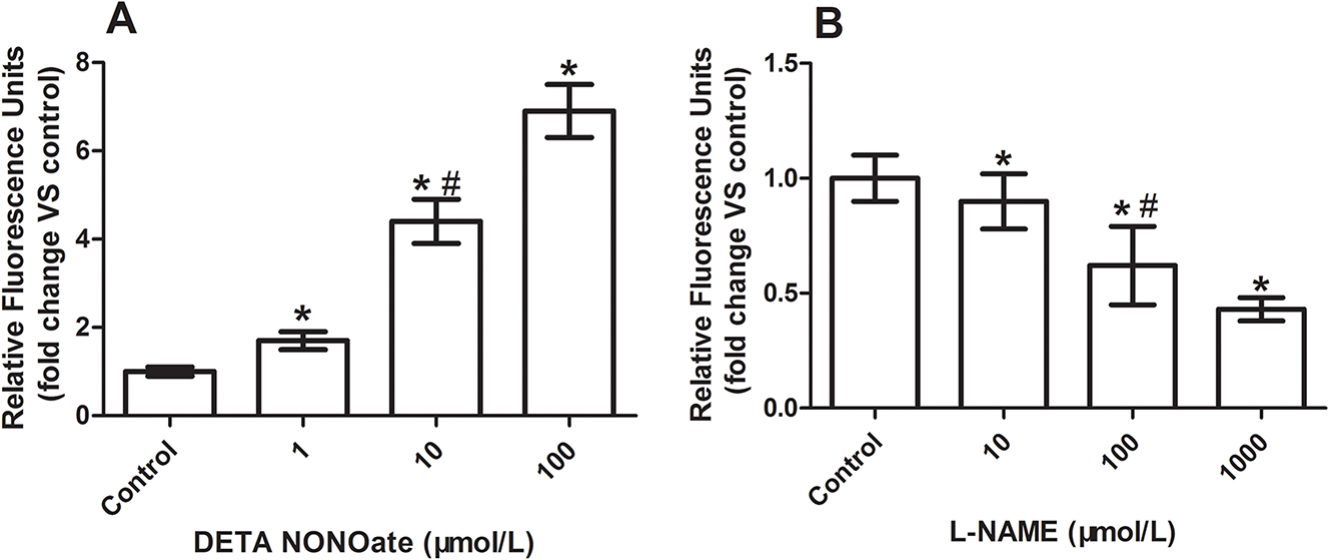
Quantitative analysis of fluorescence density (AU/μm2) of FITC-dextran in the media of cultured HUVECs treated with or without NONOate or L-NAME. A; NONOate treated. B: L-NAME treated. *P < 0.05 vs control., #P < 0.05 vs 1 or 100 μM group in A, vs 10 or 1000 μM group in B.

**Fig 10:**
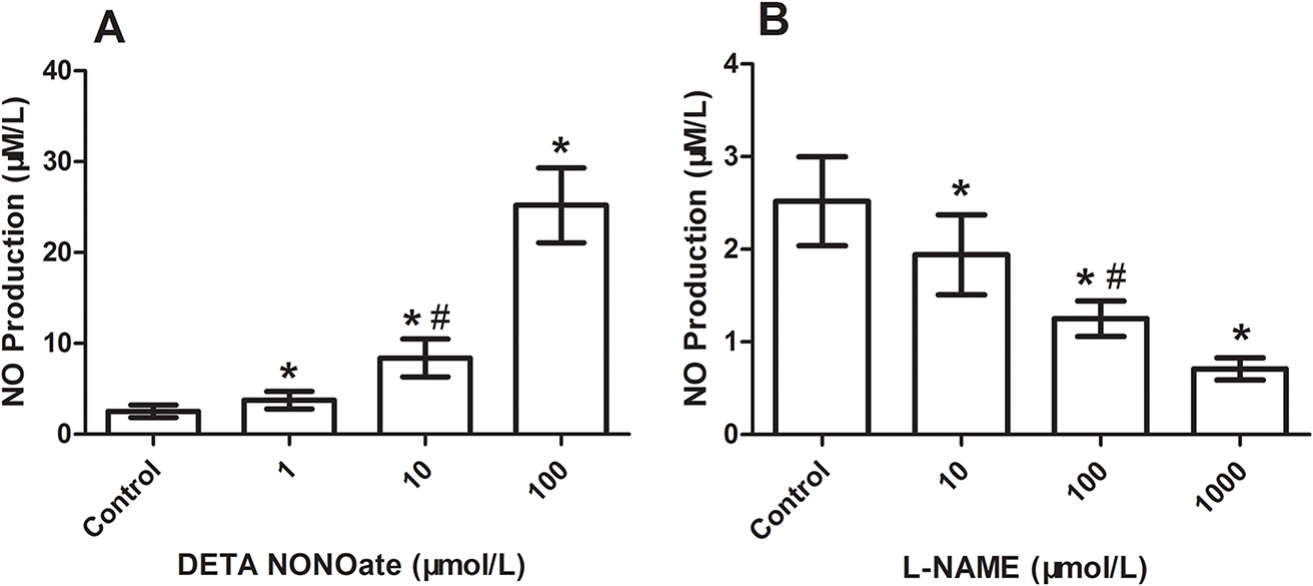
Quantitative analysis of NO production (μM/L) in the media of cultured HUVECs treated with different doses of NONOate or L-NAME. A; NONOate treated. B: L-NAME treated. *P < 0.05 vs control., #P < 0.05 vs 1 or 100 μM group in A, vs 10 or 1000 μM group in B.

To verify the effect of NONOate or L-NAME on expression of VE-cadherin and vascular permeability observed during arteriogenesis, we investigated the effects of NONOate or L-NAME on the expression of VE-cadherin and the changes of EC permeability in cultured HUVECs. The VE-cadherin was determined by immunofluorescence and immunoblotting. EC permeability was checked by FITC-dextan leakage in transwell inserts system. In untreated HUVECs, VE-cadherin staining was continuous and distributed around the entire periphery of cells (Fig 8A). Exposed to NONOate (100 μmol/L) for 24 h, the VE-cadherin staining became intermittent, showing frequent gaps (Fig 8B), the amount of VE-cadherin protein was significantly decreased (Fig 8D), which was further confirmed by immunoblotting (Fig 8E). In contrast, exposed to L-NAME (1000 μmol/L) for 24 h, VE-cadherin staining was strong, continuous and distributed around the entire periphery of cells (Fig 8C). The amount of VE-cadherin protein was even significantly higher than that in controls (Fig 8D and 8F). FITC-dextran leakage was low in untreated HUVECs, but significantly increased after exposed to NONOate, which was dose-dependent (Fig 9A). In contrast, FITC-dextran leakage was significantly reduced in L-NAME treated HUVECs (Fig 9B). Colorimetric Assay confirmed that NO production was significantly increased in HUVECs exposed to NONOate, but significantly reduced in L-NAME treated HUVECs (Fig 10).

## Discussion

The main findings of this study are: (1) VE-cadherin was down-regulated and vascular permeability was increased during arterogenesis induced by ligation of the femoral artery in rats; (2) administration of NONOate decreased VE-cadherin expression and increased vascular permeabitity, whereas L-NAME increased VE-cadherin expression and reduced vascular permeabitity during arteriogenesis; (3) In vitro experiments demonstrated that exposure of HUVEs to NONOate led to a decrease in VE-cadherin expression and an increased in FITC-dextran leakage, whereas L-NAME increased Ve-cadherin expression in HUVEs and prevented FITC-dextran leakage.

The relationship bewteen vascular permeability and angiogenesis has been well recognized. On one hand, increased vascular permeability is one of the initial events during angiogenesis[19]; on the other hand, rapid angiogenesis is accompanied by increased vessel permeability[20]. Previous studies showed arteriogenesis was accompanied by an inflammation event in which monocytes/macrophages transmigrate through the endothelial barrier, invade into deeper parts of the vessel wall, and/or populate the adventitial space [1,3,4,5]. An intuitive consequence of these observations is that there is an increase of arterial EC permeability during arteriogensis. In this study, we found in normal arterioles, staining for VE-cadherin was intense and continuously distributed along the cell-cell borders of endothelial cells. Similar staining pattern was also observed in static confluent HUECs. In contrast, in growing collateral vessels, staining for VE-cadherin was weak and punctate along the cell-cell borders of endothelial cells. Considering the results of others that VE-cadherin plays a pivotal role in the control of vascular permeability [9–12], the finding of decreased expression and altered distribution of VE-cadherin suggests that endothelial barrier is disrupted and vascular permeability is increased during arteriogenesis. To further assess vascular permeability, FITC-conjugated dextran was injected into the tail vein of sham and femoral artery ligated rats 2 hours before sarcrifice. We found that in normal arterioles, there was no FITC-dextran leakage, but this leakage happened in growing collateral vessels. Consistent with FITC-dextran leakage, infiltration of monocytes/macrophages was rarely observed in normal arterioles, but once again confirmed in growing collateral vessels. These observations are in support of the conception that arterial EC permeability is increased that plays an important role in the recruitment of monocytes/macropjhages to the collateral vessels. To our knowledge this is the first report showing the change of arterial EC permeability during arteriogenesis. However, the other mechanism for the formation of inflammation during arteriogenesis could not be ruled out, for example, most recently Bruce et al showed that monocytes are recruited from venules during arteriogenesis in the murine spinotrapezius ligation model[21]. In fact, we also found an increase in FITC-dextran leakage took place in the capilaries in the musculus gracilis after femoral artery ligation.

Arteriogenesis is initiated by increased shear stress which is achieved once chronic occlusion of a major artery or stenosis is formed [22]. Shear stress, the tractive frictional force exerted by flowing blood on the ECs, modulates many physiological, biochemical, and molecular responses in in vitro and in vivo experiments[23,24,25,26], including endothelial permeability [27]. The endothelial permeability is mainly determined by VE-cadherin. Therefore, we speculate that decreased expression and altered distribution of VE-cadherin in growing collateral vessels could be associated with increased shear stress duting arteriogenesis. In support of this, ample evidence from in vitro and in vivo investigations showing shear stress has an impact on the localization and expression of VE-cadherin[28,29,30], for example, Noria et al showed VE-cadherin staining was intense and continuous along cell borders in confluent porcine aortic endothelial cells under static conditions, but after 8.5 hours of shear stress, VE-cadherin expression was decreased and staining was punctate [28]. Furthermore, Miao et al demonstrated that in a local stenosis model, at the site of the constriction where the wall shear stress was greatly enhanced, the cells were elongated and VE-cadherin expression at cell borders was markedly reduced[30]. However, it should be pointed out that current data about the effect of shear stress on VE-cadherin expression in in vitro experiments seems to be controversial: in Ukropec and Miao’ studies, shear stress did not down-regulate VE-cadherin expression, but induced VE-cadherin redistribution[29,30]. Whether this controversial result is due to different shear rates used in their experiments or implies more complicated mechanism is involved in shear-mediated VE-cadherin expression remains to be determined.

NO is a well-documented modulator of endothelial cell permeability in vivo and in vitro. Yet the effect of nitric oxide on arterial endotheial permeability during arteriogenesis is not reported. We previously showed that administration of the nitric oxide donor DETA NONOate strongly induced a considerable increase of mononuclear cells perivascular to growing collaterals, whereas blockage of all sources of nitric oxide reduced monocyte accumulation[16]. These observations suggest that nitric oxide may mediate vascular permeability and monocyte/macrophage invasion during arteriogenesis. Since VE-cadherin plays a pivotal role in endothelium integrity and in the control of vascular permeability[13] and endothelial nitric oxide synthase activity is linked to its presence at intercellular junctions[31], we therefore postulated that the change of VE-cadherin expression may be involved in NO-mediated changes of vascular permeability and monocyte/macrophage invasion during arteriogenesis. This hypothesis was proved by the observation in present study that an increase in NO by administration of NO donor DETA NONOate to femoral-occluded rats decreased the expression of VE-cadherin, increased FITC-dextran leakage and enhanced infiltration of monocytes/macrophages, whereas blockage of NO production with the nonspecific NOS inhibitor L-NAME abolished above phenomena. Furthermore, in in vitro experiments we also demonstrated that NO greatly alters organization and expression of VE-cadherin and endothelial permeability, similar to that seen in in vivo experiment. In support of our data, González et al reported that exposure of postconfluent microvascular endothelial cells to NO donors reduced the amount of VE-cadherin, which was correlated to increases in permeability both in vitro and in vivo[15]. In addition to the role in mediating VE-cadherin expression, NO derived from eNOS can regulate VEGF-induced stress fiber formation and VE-cadherin phosphorylation[32] and nitrosylates b-catenin, which decreases its association to VE-cadherin to promote the disruption of intercellular contacts and increase endothelial permeability[33]. Whether these mechanisms are also involved in regulating vascular permeability during arteriogenesis remains to be determined.

It is well documented that NO production is increased during arteriogenesis. We previously reported that eNOS expression was increased in growing collateral vessels, which was accompanied with phosphoration of this enzyme, indicating an increase of this enzyme activity and production of NO[18,34]. However, the role of NO in arteriogenesis seems to be a controversial issue. Targeted disruption of eNOS does not prevent arteriogenesis[35], whereas the NO synthase inhibitor L-NAME abrogates shear stress-induced growth of peripheral collateral arteries[36]. Recently in the mice with targeted deletion of eNOS and of inducible nitric oxide synthase (iNOS), we demonstrated that only iNOS knockout could partially inhibit arteriogenesis, but the combination of eNOS knockout and treatment with the iNOS inhibitor L-NIL completely abolished arteriogenesis[16]. Furthermore, we also showed that besides eNOS, iNOS mRNA became upregulated in shear stress-stimulated collateral vessels[16], suggesting that increased iNOS could be one important mechanism for arteriogenesis. It was reported that inflammatory cells such as macrophages also contribute to iNOS production[37]. In present study, using immunostaining we showed iNOS protein was mainly localized at smooth muscle cells, but also observed in macrophages. Therefore we speculate that increased NO in growing collateral vessels could be one important mechanism for opening the entry of macromolecules and immune cells into the vascular wall, on one hand, NO from eNOS or /iNOS of endothelial cells induces the disruption and loss of the VE-cadherin complex; on the other hand, at the sites where monocytes adhere to, additional NO released from these monocytes would further weaken intercellular contact to facilitate their own invasion. It should be pointed out that although by now it is known that monocytes are not a homogenouse cell population and that there are numerous subsets of circulating monocytes and/or activated macrophages, the present study mainly focused on the relationship among monocytes/macrophages-NO-VE cadherin. However, further work is needed to determine which subpopulation of monocytes/macrophages are targeted by the NO-VE Cadherin axis.

Most recently, Li et al showed that vitronectin increases vascular permeability by promoting VE-cadherin internalization at cell junctions[38]. We previously showed that u-PA, fibronectin, vitronectin and αvβ3 integrin are upregulated during arteriogenesis [39,40], implying vitronectin-αvβ3 integrin pathway might also be associated with VE-cadherin function in growing collateral vessels. However, the finding that the supplement of NO donor DETA NONOate decreases the amount of VE-cadherin and NO inhibitor L-NAME keeps VE-cadherin at a high level strongly suggests that NO plays an important part in mediating the organization and expression of VE-cadherin.

In conclusion, our data for the first time reveal the expression profile of VE-cadherin and alterations of vascular permeability during arteriogenesis, and suggest that NO-mediated VE-cadherin pathway may be one important mechanism responsible, at least in part, for macrophage invasion during arteriogenesis.

## Supporting Information

S1 Fig Quantitative analysis of immunoblotting density of VE-cadherin in control, NONOate treated, L-NAME treated HUVECs. HUVECs grown close to confluence were pretreated with NONOate (100 μmol/L) or L-NAME (1000 μmol/L) for 24 h. The expression of VE-cadherin protein was assessed by Western blotting. Cell lysates were subjected to 8% SDS-PAGE, transferred to nitrocellulose membrane and probed with the rabbit anti-VE-cadherin or the mouse anti-GAPDH (control protein) antibodies. In the result, exposed to NONOate, the amount of VE-cadherin protein was decreased, while exposed to L-NAME, the expression of VE-cadherin protein was higher than that in controls. *P < 0.05 vs sham.
(TIF)

S1 Table The data of fluorescence density (AU/μm^2^) of FITC-dextran in the collaterals and capilaries of the musculus gracilis from sham, femoral artery ligation (FAL), NONOate treated (FAL + NONOate) and L-NAME treated (FAL + L-NAME) groups. (DOC)
