# Supplementary figures and images for: Nitric Oxide Increases Arterial Endotheial Permeability through Mediating VE-Cadherin Expression during Arteriogenesis
Source: PLoS One. 2015 Jul 2;10(7):e0127931. doi: 10.1371/journal.pone.0127931 (PMC4489889; doi:10.1371/journal.pone.0127931)

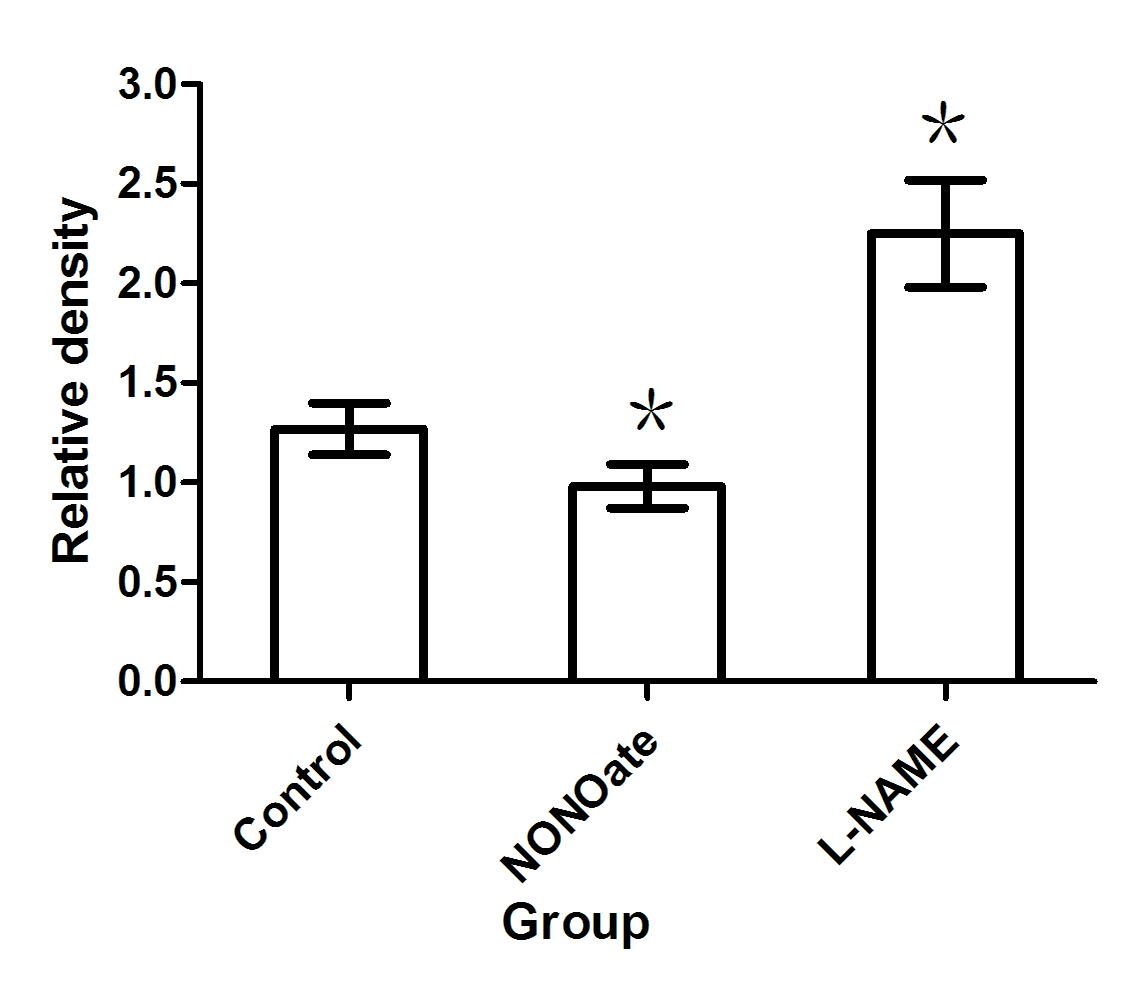

Supplement: S1 Fig — HUVECs grown close to confluence were pretreated with NONOate (100 μmol/L) or L-NAME (1000 μmol/L) for 24 h. The expression of VE-cadherin protein was assessed by Western blotting. Cell lysates were subjected to 8% SDS-PAGE, transferred to nitrocellulose membrane and probed with the rabbit anti-VE-cadherin or the mouse anti-GAPDH (control protein) antibodies. In the result, exposed to NONOate, the amount of VE-cadherin protein was decreased, while exposed to L-NAME, the expression of VE-cadherin protein was higher than that in controls. *P < 0.05 vs sham. (TIF) [file pone.0127931.s001.tif]
