# Supplementary material for: Nitric Oxide Increases Arterial Endotheial Permeability through Mediating VE-Cadherin Expression during Arteriogenesis
Source: PLoS One. 2015 Jul 2;10(7):e0127931. doi: 10.1371/journal.pone.0127931 (PMC4489889; doi:10.1371/journal.pone.0127931)
Supplement: S1 Table — (DOC) [file pone.0127931.s002.doc]

S1 Table: The data of fluorescence density (AU/µm^2^) of FITC-dextran in the collaterals and capilaries of the [musculus gracilis](http://dict.cn/musculus gracilis) from sham, femoral artery ligation (FAL), NONOate treated (FAL + NONOate) and L-NAME treated (FAL + L-NAME) groups.

| Group | Collateral vessel | Capilary |
| --- | --- | --- |
| Sham | 2.61±0.56 | 18.32±2.52 |
| FAL | 30.43±1.50^*^ | 40.77±1.81^*^ |
| FAL + NONOate | 35.28±3.12^*#^ | 50.81±2.99^*#^ |
| FAL + L-NAME | 3.55±1.18^#^ | 22.34±1.74^#^ |

*P ﹤ 0.05 vs sham., #P ﹤ 0.05 vs FAL
